# SWEET transporters of Medicago lupulina in the arbuscular-mycorrhizal system in the presence of medium level of available phosphorus

**DOI:** 10.18699/VJGB-23-25

**Published:** 2023-06

**Authors:** A.A. Kryukov, A.O. Gorbunova, T.R. Kudriashova, O.B. Ivanchenko, M.F. Shishova, A.P. Yurkov

**Affiliations:** All-Russia Research Institute for Agricultural Microbiology, Pushkin, St. Petersburg, Russia; All-Russia Research Institute for Agricultural Microbiology, Pushkin, St. Petersburg, Russia; All-Russia Research Institute for Agricultural Microbiology, Pushkin, St. Petersburg, Russia Peter the Great St. Petersburg Polytechnic University, St. Petersburg, Russia; Peter the Great St. Petersburg Polytechnic University, St. Petersburg, Russia; Saint Petersburg State University, Biological Faculty, St. Petersburg, Russia; All-Russia Research Institute for Agricultural Microbiology, Pushkin, St. Petersburg, Russia

**Keywords:** arbuscular mycorrhiza, Medicago lupulina, Rhizophagus irregularis, SWEET, gene expression assessment, sugar transporter genes, арбускулярная микориза, Medicago lupulina, Rhizophagus irregularis, SWEET, оценка экспрессии генов, гены транспортеров сахаров

## Abstract

Arbuscular mycorrhiza (AM) fungi receive photosynthetic products and sugars from plants in exchange for contributing to the uptake of minerals, especially phosphorus, from the soil. The identification of genes controlling AM symbiotic efficiency may have practical application in the creation of highly productive plant-microbe systems. The aim of our work was to evaluate the expression levels of SWEET sugar transporter genes, the only family in which sugar transporters specific to AM symbiosis can be detected. We have selected a unique “host plant–AM fungus” model system with high response to mycorrhization under medium phosphorus level. This includes a plant line which is highly responsive to inoculation by AM fungi, an ecologically obligate mycotrophic line MlS-1 from black medick (Medicago lupulina) and the AM fungus Rhizophagus irregularis strain RCAM00320, which has a high efficiency in a number of plant species. Using the selected model system, differences in the expression levels of 11 genes encoding SWEET transporters in the roots of the host plant were evaluated during the development of or in the absence of symbiosis of M. lupulina with R. irregularis at various stages of the host plant development in the presence of medium level of phosphorus available for plant nutrition in the substrate. At most stages of host plant development, mycorrhizal plants had higher expression levels of MlSWEET1b, MlSWEET3c, MlSWEET12 and MlSWEET13 compared to AM-less controls. Also, increased expression relative to control during mycorrhization was observed for MlSWEET11 at 2nd and 3rd leaf development stages, for MlSWEET15c at stemming (stooling) stage, for MlSWEET1a at 2nd leaf development, stemming and lateral branching stages. The MlSWEET1b gene can be confidently considered a good marker with specific expression for effective development of AM symbiosis between M. lupulina and R. irregularis in the presence of medium level of phosphorus available to plants in the substrate.

## Abstract (ru)

Грибы арбускулярной микоризы (АМ) помогают растениям усваивать из почвы минеральные вещества, особенно фосфор, в то время как получают от растений продукты фотосинтеза – сахара. Выявление генов, контролирующих симбиотическую эффективность АМ, может иметь практическое применение при создании высокопродуктивных растительно-микробных систем. Поэтому целью работы была оценка уровней экспрессии генов семейства транспортеров сахаров SWEET – единственного семейства, где могут быть обнаружены специфические для АМ-симбиоза транспортеры сахаров. Нами разработана уникальная высокочувствительная к уровню фосфора и микоризации модельная система «растение-хозяин–АМ-гриб», включающая высокоотзывчивую на инокуляцию АМ-грибами экологически облигатно микотрофную линию MlS-1 люцерны хмелевидной (Medicago lupulina) и штамм АМ-гриба RCAM00320 Rhizophagus irregularis, обладающий высокой эффективностью на множестве видов растений. С использованием этой модельной системы в корнях растения оценены изме- нения в уровне экспрессии 11 генов, кодирующих транспортеры семейства SWEET, при развитии либо при от- сутствии симбиоза M. lupulina с R. irregularis в различные фазы развития растения-хозяина в условиях средне- го уровня доступного для питания растений фосфора в субстрате. У микоризованных растений обнаружены более высокие уровни экспрессии в большинстве фаз развития растения-хозяина для MlSWEET1b, MlSWEET3c, MlSWEET12, MlSWEET13 по сравнению с контролем без АМ. Повышенная относительно контроля экспрессия при микоризации наблюдалась также и для MlSWEET11 в фазу развития 2-го и 3-го листьев, для MlSWEET15с – в фазу стеблевания, для MlSWEET1а – в фазу 2-го листа, стеблевания и бокового ветвления. Ген MlSWEET1b с уверенно- стью можно считать хорошим маркером со специфической экспрессией для эффективного развития АМ-симбиоза M. lupulina с R. irregularis в условиях среднего уровня доступного для растений фосфора в субстрате

## Introduction

Plant sugar transporters belong to three key families: Sucrose Transporters (SUT = SUC), Monosaccharide Transporters (MST, including subfamilies STP, TMT, PMT, VGT, pGlct/ SGB1, ESL, INT) and Sugars Will Eventually be Exported Transporters (SWEET). SUT transporters are involved in loading the phloem with sucrose and its long-distance transport from the leaves to other parts of the plant. There sugars are broken down into monosaccharides and transported to the cells by MST proteins

The least studied of these groups is the SWEET family of transporters. These are non-volatile and bidirectional transmembrane transporters of various sugars in all plant organs and tissues. L.Q. Chen’s was first to account for this family of transporters in 2010. It is in the SWEET family that the proteins particular to AM-symbiosis could be identified, while ones in the other two families had not yet been found (Chen et al., 2010; Doidy et al., 2019). Currently, it is believed that SWEET transporters are found in all living organisms (Feng et al., 2015). At the same time, it is noted that the number of isoforms of these transporters differs even in closely related species. The numbering of new SWEET proteins and their isoforms in other organisms is executed according to gene orthology principals as with the proteins in Arabidopsis thaliana.

As a result of early studies of transporters, it turned out that the SWEET plant genes, despite their low homology, are grouped into four clades (Chen et al., 2015). Representatives of each of the clades are observed in almost all terrestrial plants. It is believed that the representatives of the four clades are phylogenetically and functionally distinct. Thus, it is noted that representatives of clades I and II transport hexoses, clade III, mainly sucrose, and clade IV, mainly fructose (Chen et al., 2012; Feng et al., 2015).

SWEET proteins are involved in a variety of processes. In addition to the transport of sugars, they apparently participate in the transport of other substances, for example, gibberellins, as was shown for Arabidopis (Kanno et al., 2016). A great deal of data in the literature deals with the functions of SWEET proteins in different plant species. For example, the MtSWEET1b transporter may supply glucose to AM fungi (An et al., 2019), LjSWEET3 mediates sucrose transport (Sugiyama et al., 2017) to nodules of Lotus japonicus. SWEET clade I transporters are probably involved in the supply of sugars to symbiotic systems (Doidy et al., 2019). Rhizosphere pathogens can cause increased synthesis of clade III proteins. This, in turn, leads to additional sucrose transport to roots and contributes to the nutrition of microorganisms in the rhizosphere (Doidy et al., 2019). In 2010, L.Q. Chen et al. demonstrated that pathogenic bacteria, for example, of the genus Xanthomonas, can enter tissues of the host plant and induce expression of the SWEET genes. These encode transporters from clade III (primarily SWEET11 and SWEET14) in order to produce sugars. Like symbiotic AM fungi, pathogenic fungi induce gene expression to produce sugars (Chen et al., 2010).

J. Manck-Gotzenberger and N. Requena (2016) note that the genes of many transporters have a significant level of expression in AM symbiosis, but are not necessarily particular to it. The work of A. Kafle shows that the orthologs of SWEET1 – MtSWEET1.2 and PsSWEET1.2 – can be expressed both in mycorrhizal roots and in root nodules (Kafle et al., 2019). Therefore, the orthologs of the transporters of clades I (MtSWEET1-MtSWEET3) and III (MtSWEET9- MtSWEET15) are primarily considered as active participants in the symbiotic relationship “plant–AM fungus” (Kryukov et al., 2021).

The study of the role of SWEET transporters in the formation of symbiotic relationships has never been specifically directed toward PMS. In this regard, the aim of this work was to evaluate the expression of the SWEET genes in PMS models during mycorrhization and its respective absence at different stages of plant development

## Materials and methods

Plant and fungal material. Black medick (Medicago lupulina L. subsp. vulgaris Koch) is a widespread species of the genus Medicago, a self-pollinating diploid. In the present study, the authors selected the MlS-1 line as being highly responsive to mycorrhization from the black medick cultivar-population VIK32 (Yurkov et al., 2015). Barring inoculation by AMfungus, and with low level of available inorganic phosphorus (Pi) in the soil, this line exhibits signs of dwarfism (Yurkov et al., 2015, 2020). An effective strain RCAM00320 of the AM fungus Rhizophagus irregularis (formerly known as Glomus intraradices Shenck & Smith) was used for inoculation (CIAM8 from the All-Russia Research Institute for Agricultural Microbiology (ARRIAM) collection). An accurate identification of the strain was carried out by the authors (Kryukov, Yurkov, 2018). Since AM-fungi are obligate symbionts, the strain is maintained in a cumulative culture of Plectranthus sp. (exact species identification is currently being undertaken by the authors) under standard conditions in the ARRIAM Laboratory of ecology of symbiotic and associative microorganisms (Yurkov et al., 2010).

Vegetative method. The procedure for the method is described in the work of A.P. Yurkov et al. (Yurkov et al., 2015). Optimal conditions were provided for the development of AM while preventing spontaneous infection with nodule bacteria and other microorganisms. A mixture of soil and sand in a ratio of 2:1 was autoclaved twice at 134 °C, 2 atm for 1 hour with repeated autoclaving two days later; no signs of toxicity appeared after such treatment. Specimens were planted with two seedlings per one vessel filled with a soil-sand mixture (210 g). Agrochemical characteristics of the soil are given by (Yurkov et al., 2015). The content of P2O5 in the soil was 23 mg/kg of soil (as according to Kirsanov). Before the experiment, 0.5 doses of phosphorus were added in the form of CaH2PO4*2H2O (86 mg/kg of soil) according to the prescription of D.N. Pryanishnikov (Klechkovsky, Petersburg-sky, 1967). The final phosphorus content in the soil-sand mixture was 109 mg/kg and corresponded to the average Pi level; i. e. the availability of mobile phosphates in the soil in terms of the content in the Kirsanov extract according to (Sokolov, 1975); pHKCl – 6.44. The first measurement of plants was carried out 21 days after sowing and inoculation, followed by measurement at key stages of black medick ontogenesis. There was a total number of 7 measurements (Supplementary Material 1)1.

Supplementary Materials are available in the online version of the paper: http://vavilov.elpub.ru/jour/manager/files/Suppl_Kryukov_Engl_27_3.pdf.


The specimens involved Plectranthus roots inoculated and uninoculated with R. irregularis strain RCAM00320. During collection, the material was frozen in liquid nitrogen and stored for up to 6 months at –80 °C.

RNA isolation and evaluation of gene expression. The selection of genes of interest was carried out based on the results of M. truncatula transcriptome analysis (MtSWEET1a = Medtr1g029380, MtSWEET1b = Medtr3g089125, MtSWEET2a = Medtr8g042490, MtSWEET2b = Medtr2g073190, MtSWEET2c = Medtr6g034600, MtSWEET3a = Medtr3g090940, MtSWEET3b = Medtr3g090950, MtSWEET3c = Medtr1g028460, MtSWEET4 = Medtr4g106990, MtSWEET5a = Medtr6g007610, MtSWEET5b = Medtr6g007637, MtSWEET5c = Medtr6g007623, MtSWEET5d = Medtr6g007633, MtSWEET6 = Medtr3g080990, MtSWEET7 = Medtr8g099730, MtSWEET9a = Medtr5g092600, MtSWEET9b = Medtr7g007490, MtSWEET11 = Medtr3g098930, MtSWEET12 = Medtr8g096320, MtSWEET13 = Medtr3g098910, MtSWEET14 = Medtr8g096310, MtSWEET15a = Medtr2g007890, MtSWEET15b = Medtr5g067530, MtSWEET15c = Medtr7g405730, MtSWEET15d = Medtr7g405710, MtSWEET16 = Medtr2g436310; sequence numbers from the database: https://phytozome.jgi.doe.gov/pz/portal.html) with subsequent selection of primer sequences for the genes of interest.

Three pairs of primers were tested for each gene. The absence of the second product was estimated based on electrophoresis and melting curves. The effectiveness of primers was calculated on the basis of real-time PCR (quantitative poly merase chain reaction in real time) serial dilutions of the cDNA matrix. Only primers with efficiency equal to or close to 100 % were used. The primer test was carried out for several measurement periods (Supplementary Material 2).

In 2022, the conformity and quality of primers were verified using M. lupulina MlS-1 transcriptomic data (MACE sequencing). Total RNA from plant material was isolated using the trizole method with modifications (MacRae, 2007). The quality of DNAase treatment was tested by PCR for RNA with the reference gene, actin tested immediately before cDNA synthesis. cDNA synthesis was carried out using the Maxima First Strand cDNA Synthesis Kit with dsDNase in accordance with the manufacturer’s instructions (Thermo Scientific, USA). ~1 mcg of total RNA was selected for cDNA synthesis. cDNA quality was tested with a ubiquitin test.

Changes in gene expression were evaluated using the RT-PCR method employing the BioRad CFX-96 real-time thermal cycler (Bio-Rad, USA) and using a set of reagents for RT-PCR in the presence of the SYBR Green I dye. The parameters of the amplification cycles were as follows: 95 °C, 5 min, 1 cycle; 95 °C, 15 s, 60 °C, 30 s, 72 °C, 30 s, 40 cycles. The specificity of amplification was evaluated using melting curve analysis. Changes in the expression level of the gene under examination were compared with the expression level of the same gene in the control. Analysis was carried out using the 2–ΔΔCT method. The levels of gene expression were normalized with the selected reference gene, actin according to (Yurkov et al., 2020). The PCR mix (10 ml) contained: 1 ml of 10x B + SYBR Green buffer, 1 ml of 2.5 mM dNTP, 1 ml of MgCl2 (25 mM), 0.3 ml of each of a pair of primers (10 mM for each primer), 0.125 ml (0.625 units) SynTaq DNA polymerase (manufacturer of mix components – Synthol, Russia), 4.275 μl ddH2O, 2 μl cDNA sample. The relative values of the cDNA gene expression level for each sample were evaluated (experiment with AM, control without AM). The biological repeatability is 3, the technical repeatability is 4 measurements.

Evaluation of parameters of symbiotic efficiency and activity. J.M. Philips and D.S. Hayman’s trypan blue staining method was used for root samples (Phillips, Hayman, 1970). The parameters of AM fungus activity in the root, the mycorrhization indices, are calculated according to (Vorob’ev et al., 2016) as: a and b (abundance of arbuscules and vesicles in mycorrhized parts of the roots, respectively), and M (intensity of AM development in the root). They have the following calculation formulas:

**Formula. 1.**
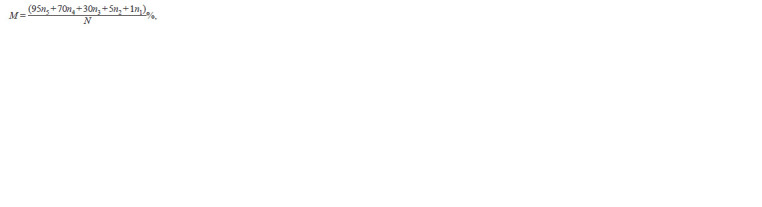
Formula. 1.

where n5 is the number of visual fields with a mycorrhiza density class – M = 5; n4 – with M = 4; n3 – with M = 3, etc.; M is estimated from 1 to 5 points: 1 evaluation score: 0–1 % mycorrhiza at the root in the field of view of the microscope; 2 evaluation scores: 2–10 %; 3 scores: 11–50 %; 4 scores: 51–90 %; 5 scores: 91–100 % mycorrhiza in the root

**Formula. 2.**
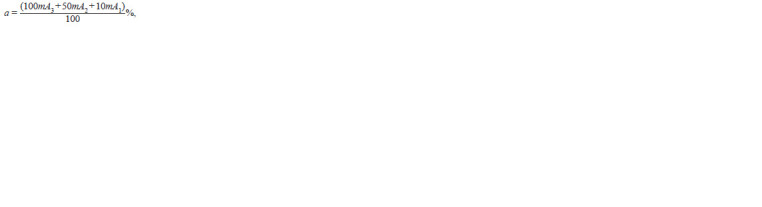
Formula. 2.

where mAi =

**Formula. 3.**
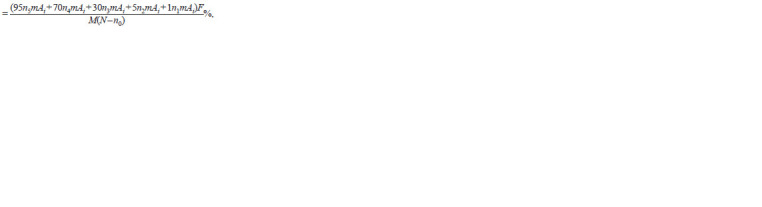
Formula. 3.

where ni mAj is the number of visual fields with M = i, A = j, F is the incidence of mycorrhizal infection (the proportion of visual fields with AM relative to the total number of visual fields in one root sample), N is the total number of viewed visual fields, n0 is the number of visual fields without AM, ni is the number of visual fields with a mycorrhiza density class from 1 to 5, and Ai is the arbuscule density class from 1 to 3.

**Formula. 4.**
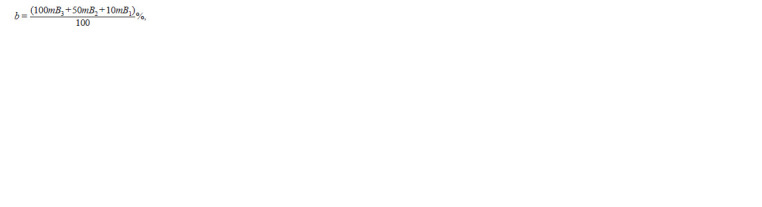
Formula. 4.

where ni mBj is the number of visual fields with M = i and B (vesicle density class) = j is calculated similarly to the calculation for arbuscules (3); Bj is the density class of arbuscules from 1 to 3.

The symbiotic efficiency of AM was estimated as the difference in productivity index (crude weight of aboveground parts) between the variant with AM inoculation (“+AM”) and the control without AM (“without AM”), divided by the value in the variant “without AM” as a standard calculation MGR (mycorrhizal growth response) (Kaur et al., 2022). Biological repeatability in assessing the parameters of the effectiveness and activity of AM in each variant was equal to 8 plants.

Statistical analysis. ANOVA and Tukey’s HSD test ( p < 0.05) were used as a post-hoc test to compare the differences in all indicators; Student’s t-test ( p < 0.05) was also used to assess the significance of differences in the average values of gene expression levels between the “+AM” and “without AM” variants

## Results

The results of the evaluation of the symbiotic efficacy and parameters of mycorrhization showed that the studied symbiotic test system Medicago lupulina + Rhizophagus irregularis should be considered highly effective (with high MGR) and symbiotically active. Symbiotically active refers to the presence of active mycorrhization of roots by mycelium, arbuscules and vesicles under conditions of an average level of phosphorus available to plants in the substrate

**Fig. 1.**
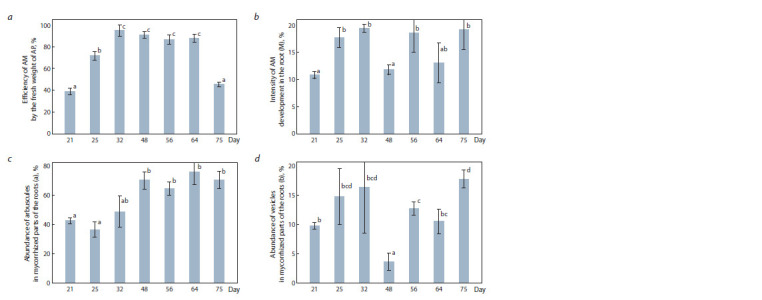
The symbiotic efficiency of AM calculated from the fresh weight of the aboveground parts (a), the intensity of mycorrhization M (b), the abundance of arbuscules (c) and the abundance of vesicles (d ) formed by R. irregularis in the roots of M. lupulina. The average values with the error of the average are presented; the LF is the aboveground parts; “day” is the day after sowing and inoculation. Values with significant (p < 0.05) differences are marked with different letters (a, b, c, d).

Analysis of M. lupulina mycorrhization by AM fungus R. irregularis showed that the intensity of mycorrhization (M; Fig. 1, b) and the abundance of vesicles (b, see Fig. 1, d ) significantly decreased at the initiation of lateral branching stage (48 days). However the abundance of arbuscules (a, see Fig. 1, c), the principal symbiotic structures of AM, were maintained at a high level along with the symbiotic efficiency (MGR) calculated for the fresh weight of the aboveground parts (see Fig. 1, a).

**Fig. 2.**
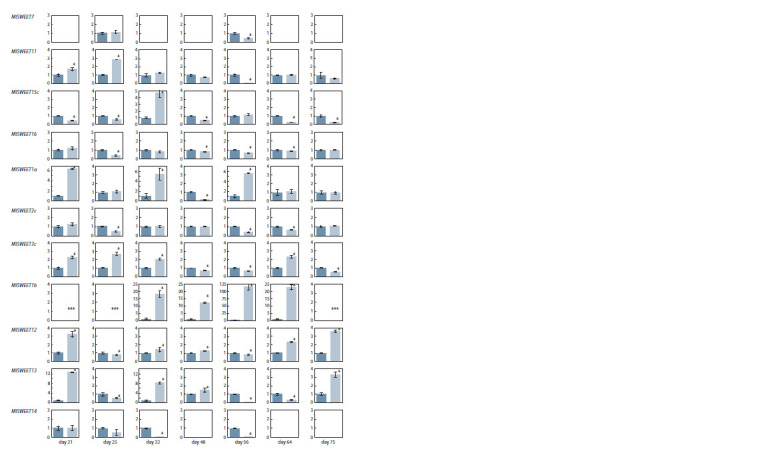
Relative transcript level (normalized value of 2–ΔΔCt) of the SWEET family genes in the roots of M. lupulina. The average values with the error of the average are presented. * The presence of significant ( p < 0.05) differences between the variant “without” (dark bars) and the variant “+AM” with R. irregularis inoculation (light column); empty diagrams – the absence of gene expression in the variants; “day” – day after sowing and inoculation. *** Specific gene expression in the variant “with AM”).

The obtained data on microscopy and MGR evaluation indicate that highly effective and active PMS with an early and prolonged response can be used as a genetic model for the search and analysis of marker genes for the development of effective AM symbiosis. It can be accessed from the early stage (2nd leaf stage) to the late stage of the fruiting initiation in conditions of an average Pi level in the substrate. To this end, the expression of 11 genes of the SWEET family was evaluated. The relative level of transcripts (normalized values of 2–ΔΔCt) in the roots of M. lupulina with normalization to control without AM was evaluated (Fig. 2).

## Conclusion

The expression of the genes of the SWEET family during the transition of plants from the initiation of one stage of development to another has been practically ignored in the literature. This study has managed to eliminate this drawback. For the first time the analysis of their expression in the roots of an M. lupulina line highly responsive to mycorrhization was performed under conditions of average Pi level in the substrate. Results showed that the expression of the MlSWEET1b gene specifically increased with a decrease in symbiotic efficiency calculated by the weight of fresh aboveground parts. It is likely that the high expression in AM plants at early stages of development is associated with the active redistribution of sugars during the formation of effective AM. At the fruiting stage, on the other hand, it is a response to the needs of sugars for seed maturation. More than half of the studied genes also showed increased expression, among which genes such as MlSWEET3c and MlSWEET12 should be singled out. The results obtained are consistent with the literature in that AMspecific genes of the SWEET family can be found among the genes of clades I and III.

Given the diversity of orthologs in other plant species, there is reason to believe that not all the genes of the SWEET family have yet been identified, both in the plant we have examined, M. lupulina, and in other species of the Medicago genus. Research over the coming years will surely expand our conception of what functions SWEET transporters perform in Medicago plants.

## Conflict of interest

The authors declare no conflict of interest.
